# Etymologia: Sparganosis

**DOI:** 10.3201/eid2304.ET2304

**Published:** 2017-04

**Authors:** Ronnie Henry

**Keywords:** etymologia, sparganosis, Diphyllobothrium, tapeworms, cestodes, larvae, parasites, frogs, snakes, Asia, Africa

## Sparganosis [spahrʺge-noʹsis]

Sparganosis (Figure) refers to tissue infection with the pleurocercoid larvae of the genera *Diphyllobothrium* (from the Greek *di* [“two”] + *phyllon* [“leaf”] + *bothrion* [“pit”]) or *Spirometra* (from the Greek *speira* [“coil”] + *metra* [“uterus”]). *Sparganum* (from the Greek *sparganon* [“swaddling clothes”]) was originally described in 1854 by Diesing as a separate species but is now used generically to describe the larval stage of these cestodes.

**Figure:**
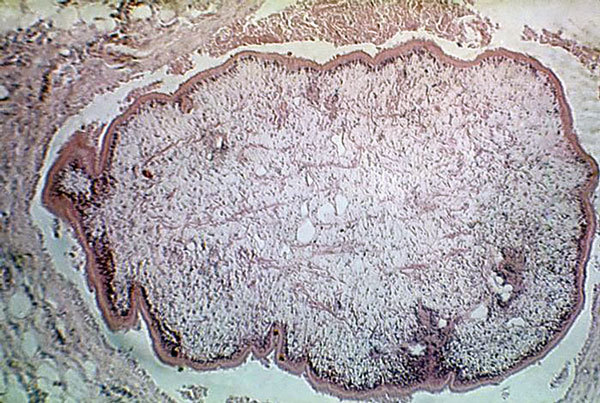
Histopathology of Sparganum proliferum infection. Public Image Health Library, Centers for Disease Control, 1962.

The first human case was reported by Sir Patrick Manson in China in 1882, and 2 species (*S. mansoni* and *S. mansonoides*) are named for him. Sparganosis is most common in Asia where frogs or snakes are more commonly eaten or where traditional medicinal practices call for the use of raw frog or snake meat in poultices, although recent reports indicate it occurs in in some populations in Africa.
